# Coordinate regulation of DNA methyltransferase expression during oogenesis

**DOI:** 10.1186/1471-213X-7-36

**Published:** 2007-04-19

**Authors:** Diana Lucifero, Sophie La Salle, Déborah Bourc'his, Josée Martel, Timothy H Bestor, Jacquetta M Trasler

**Affiliations:** 1 Montreal Children's Hospital Research Institute and Departments of Pediatrics, Human Genetics, and Pharmacology & Therapeutics, McGill University, Montreal, Quebec, H3H 1P3, Canada; 2 Department of Genetics and Development, College of Physicians and Surgeons of Columbia University, New York, New York, 10032, USA

## Abstract

**Background:**

Normal mammalian development requires the action of DNA methyltransferases (DNMTs) for the establishment and maintenance of DNA methylation within repeat elements and imprinted genes. Here we report the expression dynamics of *Dnmt3a *and *Dnmt3b*, as well as a regulator of DNA methylation, *Dnmt3L*, in isolated female germ cells.

**Results:**

Our results indicate that these enzymes are coordinately regulated and that their expression peaks during the stage of postnatal oocyte development when maternal methylation imprints are established. We find that Dnmt3a, Dnmt3b, Dnmt3L and Dnmt1o transcript accumulation is related to oocyte diameter. Furthermore, DNMT3L deficient 15 dpp oocytes have aberrantly methylated *Snrpn*, *Peg3 *and *Igf2r *DMRs, but normal IAP and LINE-1 methylation levels, thereby highlighting a male germ cell specific role for DNMT3L in the establishment of DNA methylation at repeat elements. Finally, real-time RT-PCR analysis indicates that the depletion of either DNMT3L or DNMT1o in growing oocytes results in the increased expression of the *de novo *methyltransferase *Dnmt3b*, suggesting a potential compensation mechanism by this enzyme for the loss of one of the other DNA methyltransferases.

**Conclusion:**

Together these results provide a better understanding of the developmental regulation of *Dnmt3a*, *Dnmt3b *and *Dnmt3L *at the time of *de novo *methylation during oogenesis and demonstrate that the involvement of DNMT3L in retrotransposon silencing is restricted to the male germ line. This in turn suggests the existence of other factors in the oocyte that direct DNA methylation to transposons.

## Background

DNA methyltransferases (DNMTs) catalyze the addition of methyl residues to cytosine bases within CpG dinucleotides to increase the information content of the genome. To date, DNA methylation is the best studied epigenetic modification and is found in the genomes of vertebrates, plants and fungi as well as some species of bacteria and invertebrates [1]. In mammals, the bulk of DNA methylation patterns are established during gametogenesis and early embryogenesis and as such DNA methylation is an essential developmental process. DNA methylation is important for the silencing of tandem and interspersed repeat elements and for genomic imprinting and X-chromosome inactivation [2]. Several human disorders are linked to abnormalities in DNA methylation profiles; they include cancer, Rett syndrome, ICF syndrome, as well as genomic imprinting diseases such as Angelman, Prader-Willi and Beckwith-Wiedemann syndromes [3].

DNMT enzymes are related by the well-conserved motifs in their catalytic domains and are required for two types of methyltransferase activity, *de novo *and maintenance methylation. DNMT1 is considered the major maintenance methyltransferase as it is the only DNMT known to exhibit a strong affinity for targeting hemimethylated DNA [4]. Mouse embryos made completely deficient in this enzyme through gene targeting die prior to midgestation and exhibit a 95% loss of methylation when compared to normal embryos [5]. Homozygous DNMT1-deficient embryos have hypomethylated repeat element sequences and biallelic expression of imprinted genes [6]. An oocyte-specific transcript, Dnmt1o, results from the alternative splicing of 5' sex-specific exons within *Dnmt1 *[7]. Heterozygous embryos derived from *Dnmt1o *null females show abnormalities in the methylation imprint maintenance and die pre-natally [8]. While DNMT2 is the best conserved of the eukaryotic DNMTs and possesses all the conserved motifs required for DNMT function, DNMT2 has recently been shown to specifically methylate position 38 in tRNA^Asp ^[9].

In contrast to the predominant maintenance activity of DNMT1 and DNMT1o, studies have revealed that DNMT3a and DNMT3b transfer methyl groups to hemimethylated and unmethylated substrates at roughly equivalent rates [10]. Gene targeting studies have shown that *Dnmt3a*-/- mice survive a few weeks post-natally, while *Dnmt3b*-/- embryos die prior to birth [11]. DNMT3L also belongs to the DNMT3 subfamily because of the cysteine rich motif it shares with DNMT3a and DNMT3b. However unlike any of the other DNMTs that have been characterized, DNMT3L lacks the conserved motifs necessary for enzymatic function and therefore lacks DNMT catalytic activity. Instead DNMT3L has been shown to be a potent stimulator of DNMT3a and DNMT3b by changing their conformation and favouring their binding to AdoMet and target sequences [12]. Remarkably, *Dnmt3L *gene targeting experiments have shown it to be essential for normal spermatogenesis, in particular for meiosis, and required for the methylation of DNA within the differentially methylated regions (DMRs) of imprinted genes in both oocytes and male germ cells [13-16].

Genomic imprinting refers to those genes, numbering roughly 80 to date, which are expressed exclusively from one allele in a parent of origin specific manner. The parental allele specific expression of imprinted genes requires some epigenetic modification to differentially mark the two alleles during germ cell development. Studies have revealed the majority of imprinted genes to be regulated by imprinting control elements (ICEs) which harbour allele specific DNA methylation [17]. In the male germ line, DNA methylation imprint establishment on paternally methylated genes is initiated pre-natally in prospermatogonia [18], while maternally methylated imprinted genes acquire their methylation imprint during the postnatal growth phase of oogenesis [19].

Although DNA methylation is a well studied aspect of imprinted gene regulation, until recently little has been understood about which enzymes are involved in the *de novo *methylation that marks imprinted genes during gametogenesis. While *Dnmt3L *gene targeting studies clearly showed this enzyme to be essential for the establishment of methylation imprints in both germ lines [13-16], the involvement of DNMT3a and DNMT3b in genomic imprinting remained unclear because of the poor survival and development of DNMT3a and DNMT3b null mice [11]. Conditional germ cell specific knockouts of these enzymes showed that DNMT3a, in a manner similar to DNMT3L, is essential for the *de novo *methylation of imprinted genes in male and female germ cells [20].

We have previously investigated the temporal dynamics that underlie methylation imprint establishment in oocytes [19]. Here, we examined the expression patterns of the DNMT3 family of enzymes during oocyte development in normal and DNMT-deficient mice in an effort to better understand the role these enzymes play in establishing and/or maintaining DNA methylation patterns during gametogenesis. We used real-time RT-PCR (QRT-PCR) to profile the expression of *Dnmt3a*, *Dnmt3b *and *Dnmt3L *in postnatal oocytes at five different developmental time points and investigated the influence of oocyte diameter on expression patterns. We also determined which DNMT3a isoforms were expressed in growing oocytes. To better understand the role DNMT3L plays in establishing methylation imprints, we studied the methylation profile of multiple imprinted genes in DNMT3L deficient oocytes at 15 dpp, the time point when we have previously shown the majority of DNA methylation to be established in oocytes. While DNMT3L clearly has an essential role in ensuring the methylation and silencing of retrotransposons in male germ cells, it is unknown whether it serves the same function in the female germ line. We therefore also investigated the methylation profiles of IAPs and LINE-1 elements in DNMT3L null oocytes. Finally, hypothesizing that these enzymes may be coordinately regulated during development, we monitored the effect of DNMT3L and DNMT1o depletion on the expression profiles of the other DNMTs.

## Results

### Developmental profile of Dnmt3a, Dnmt3b, and Dnmt3L during postnatal oocyte growth

We determined the expression dynamics of DNA methyltransferases in oocytes at five time points spanning the period of postnatal oocyte growth: at 5 dpp, oocytes collected were from primordial follicles that had not yet entered the growth phase and measured roughly 10–20 μm in diameter; 10 dpp represented a pool of early-growing oocytes ranging greatly from 20 to 80 μm in diameter with roughly half measuring 50 μm or less; at 15 dpp our samples consisted of mid-growing oocytes up to 80 μm in diameter with only about 5% of oocytes measuring less than 50 μm; fully grown GV stage oocytes were collected at 25 dpp; and mature ovulated metaphase II (MII) oocytes were isolated from superovulated 8 week old females.

To normalize for variations in RNA input and extraction efficiency, amplification of spiked rabbit α-globin was performed for every experiment [21]. The choice of an exogenous normalizer stemmed from the absence of genes known to be expressed at a constant level throughout oogenesis (see Additional file 1). The standard curve method was used to determine the fold changes in expression which were then calibrated to the MII oocyte sample. The QRT-PCR results plotted depict the mean from one representative experiment where each sample was repeated in triplicate.

**Figure 1:**
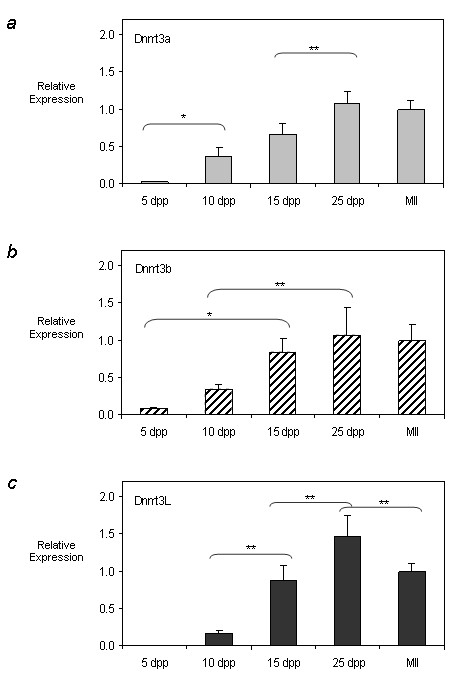
**Developmental expression profiles of *Dnmt3a*, *Dnmt3b*, and *Dnmt3L *during postnatal oogenesis**. QRT-PCR was used to determine the expression profile of **a) ***Dnmt3a *(light grey bars) **b) ***Dnmt3b *(cross hatch bars) and **c) ***Dnmt3L *(dark grey bars) in postnatal oocytes. Relative expression values obtained were normalized to the level of rabbit α-globin expression for each sample and were calibrated to the MII oocyte expression value. Representative results from one of the two independent experiments performed are plotted (see Additional file 2 for the second replicate). While the relative abundance of the transcripts varied for each of the timepoints, overall *Dnmt3a*, *Dnmt3b *and *Dnmt3L *expression increased with oocyte growth and peaked in 25 dpp oocytes for each enzyme. Results are presented as mean ± SD. * indicates significance of p < 0.05; ** indicates significance of p < 0.01.

QRT-PCR results for *Dnmt3a*, *Dnmt3b *and *Dnmt3L *are depicted in Figures 1a, b, and 1c, respectively. Expression of these transcripts increased from 5 dpp oocytes to 25 dpp oocytes, where expression peaked and then decreased slightly in MII oocytes, coinciding with the loss of transcripts which occurs in oocytes after meiotic maturation [22]. In the case of *Dnmt3a*, the relative expression at 10 dpp was significantly higher than the expression measured at 5 dpp (p < 0.05); while there was no significant difference between the same timepoints for *Dnmt3b *or *Dnmt3L*. *Dnmt3b *expression was significantly upregulated between 5 and 15 dpp (p < 0.05), suggesting a more gradual accumulation of transcripts in mid-growing oocytes (Figure 1b). The increase in *Dnmt3L *expression was highly significant between 10 dpp and 15 dpp (p < 0.01) and showed the greatest fold increase observed for any of the genes or timepoints. With respect to changes occurring during the later stages of oocyte growth, a further significant increase in expression was seen for both *Dnmt3a *and *Dnmt3L *between the 15 dpp and full grown GV stage oocytes at 25 dpp. This late increase may correlate with previously observed findings that a subset of maternally methylated imprinted genes acquires methylation late during oogenesis [19,23,24]. Another representative replicate showing similar dynamics in expression is included in the additional files (see Additional file 2).

### Relationship of oocyte diameter on Dnmt3a, Dnmt3b, Dnmt3L and Dnmt1o expression

**Figure 2:**
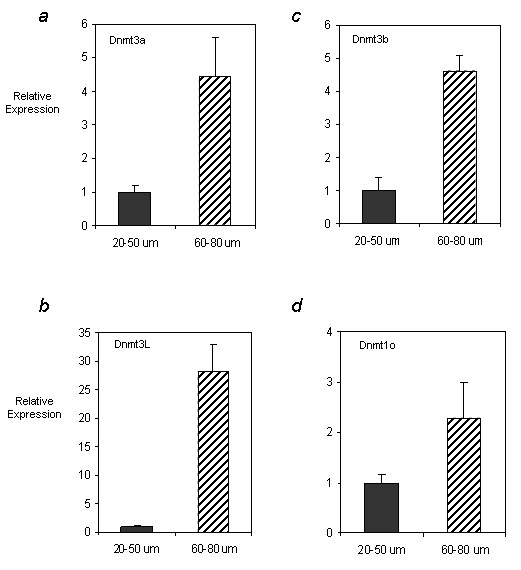
**The effect of oocyte diameter on a) Dnmt3a, b) Dnmt3b, c) Dnmt3L and d) Dnmt1o expression**. Oocytes were isolated at 10 dpp and pooled according to whether they were 20 to 50 μm or 60 to 80 μm in diameter. QRT-PCR was used to determine the relative expression of each of the enzymes in "small" (dark grey bars) vs. "big" (cross hatch bars) 10 dpp oocytes. One experiment where samples were tested in triplicate was carried out and relative expression values obtained were normalized to the level of rabbit α-globin expression for each sample and calibrated to the expression in 20 to 50 μm oocytes. For each of the enzymes investigated, the relative expression in "big" oocytes was at least 2- and up to 28-fold higher than that in "small" oocytes. Results are presented as mean ± SD. Differences between the relative expression in 20 to 50 μm and 60 to 80 μm oocytes were found to be statistically significant for each of the genes (p < 0.05).

Having previously determined that oocyte diameter at 15 dpp correlates with DNA methylation levels on the imprinted gene *Snrpn *[19], we determined whether the expression levels of *Dnmt3a*, *Dnmt3b*, *Dnmt3L *and *Dnmt1o *also changed with increasing oocyte diameter. Oocytes at 10 dpp were collected and pooled according to their diameter: "small" referring to oocytes 20 to 50 μm in diameter and "big" referring to oocytes 60 to 80 μm in diameter. For each of the *Dnmt *genes examined, 60 to 80 μm oocytes showed significantly higher (p < 0.05) levels of transcripts (Figure 2). For both *Dnmt3a *and *Dnmt3b*, relative expression levels in "big" oocytes were roughly 4 times higher than that in "small" oocytes at 10 dpp. *Dnmt1o *showed a less marked increase with oocyte diameter, with larger oocytes only showing a two-fold increase in expression. The most dramatic increase in transcript level as a result of increased oocyte diameter was seen for *Dnmt3L*, with the expression in 60 to 80 μm oocytes being 28 times that seen in 20 to 50 μm oocytes.

### DNA methylation profile of Snrpn, Peg3, Igf2r, H19, and retrotransposons IAP and LINE-1 in Dnmt3L-/- oocytes

**Figure 3:**
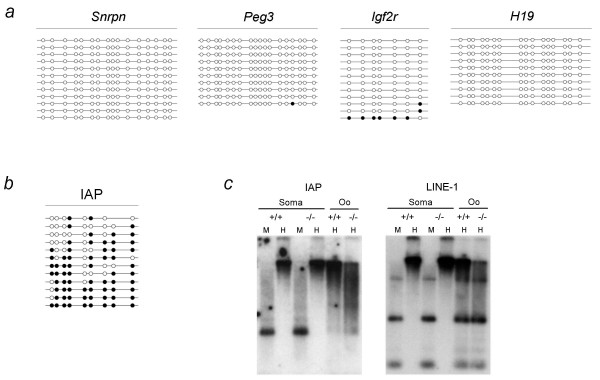
**Methylation profile of a) *Snrpn*, *Peg3*, *Igf2r*, *H19 *and b,c) IAP and LINE-1 repeats in *Dnmt3L*-/- oocytes**. **a,b) **Bisulfite sequencing results are depicted as follows: each line represents an individual clone; a filled circle indicates a methylated CpG site, an open circle denotes an unmethylated CpG and a missing circle represents a CpG site where the sequencing data were ambiguous. *H19 *methylation served as control for somatic cell contamination. While *Snrpn*, *Peg3 *and *Igf2r *appear to be abnormally unmethylated (a), IAP LTR methylation is unaffected in *Dnmt3L *homozygous oocytes at 15 dpp (b). **c) **Methylation sensitive Southern analysis of IAP LTR and LINE-1 5'UTR methylation in *Dnmt3L *+/+ and -/- cumulus cells (soma) and mature metaphase II oocytes (oo). Lanes headed by M and H contain DNA that was cleaved with MspI and HpaII, respectively. Repeat sequences appear to be normally methylated in DNMT3L depleted oocytes.

To better understand the nature of the methylation imprint defect in *Dnmt3L*-/- oocytes, we investigated the methylation profile of three maternally methylated imprinted genes and one paternally methylated imprinted gene in 15 dpp oocytes (Figure 3a). Using bisulfite sequencing, we analyzed all four genes in one pooled sample of oocytes and found the regions within the *Snrpn*, *Peg3 *and *Igf2r *DMRs sequenced to be unmethylated (analysis of *Igf2r *methylation by Fisher's exact analysis showed it to not be significantly different from 0%). To control for somatic cell contamination we determined the methylation status of *H19*, which is methylated in male germ cells but not in oocytes, and found it to be unmethylated in our 15 dpp sample. Surprisingly, although we found the maternally methylated imprinted genes to be unmethylated, CpG sites analyzed within the IAP LTR from the same set of oocytes remained normally methylated (Figure 3b). Fisher's exact analysis using wild-type IAP bisulfite data for this time point [19] showed that the CpG methylation levels obtained between wild type and DNMT3L depleted oocytes were not significantly different (p > 0.05).

Southern analysis with methylation sensitive enzymes using IAP 5' LTR and LINE-1 5' UTR probes also showed these sequences to be normally methylated in mature metaphase II oocytes therefore suggesting that in contrast to male germ cells, *Dnmt3L *is not required for establishment or maintenance of retrotransposon methylation in the female germ line (Figure 3c). While there did appear to be a slight hypomethylation of these sequences in homozygous mutant oocytes, it is important to note that these sequences are not normally fully methylated in wild-type oocytes and when compared to the dramatic differences previously observed in male germ cells [13] the overall pattern of digestion is not significantly different. Somatic cumulus cells also showed no difference in retrotransposon methylation levels (Figure 3c).

### Expression of Dnmt3 transcripts in DNMT1o and DNMT3L depleted oocytes

**Figure 4:**
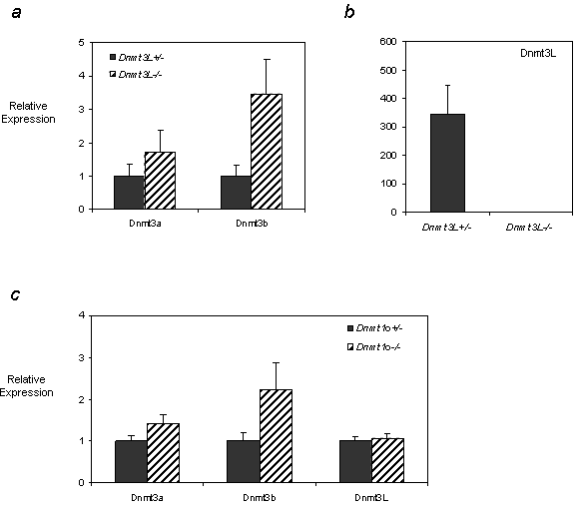
***Dnmt3a*, *Dnmt3b *and *Dnmt3L *expression in DNMT3L deficient 15 dpp oocytes and in DNMT1o deficient 25 dpp GV oocytes**. **a) **QRT-PCR was used to analyze the expression of the *de novo *DNMT enzymes in *Dnmt3L *heterozygous (dark grey bars) and homozygous (cross hatch bars) oocytes at 15 dpp. Results for *Dnmt3a *and *Dnmt3b *are shown in **a) **while relative expression of *Dnmt3L *is illustrated in **b)**. Dnmt3a and Dnmt3b transcripts are up-regulated in DNMT3L depleted growing oocytes. The differences observed for Dnmt3b and Dnmt3L were statistically significant (p < 0.01). **c) **QRT-PCR was used to analyze the expression of the DNMT enzymes in *Dnmt1o *heterozygous (dark grey bars) and homozygous (cross hatch bars) 25 dpp GV stage oocytes. While the relative expression of *Dnmt3a *and *Dnmt3b *was up-regulated in DNMT1o depleted GV oocytes, *Dnmt3L *expression remained unchanged. Samples were analyzed in triplicate and relative expression values obtained were normalized to the level of rabbit α-globin expression for each sample and were calibrated to the expression in heterozygous oocytes for *Dnmt3a*, *Dnmt3b *and *Dnmt3L*. For *Dnmt3L *analysis, expression was calibrated to the expression in homozygous oocytes. Results for one experiment are presented as mean ± SD.

In addition to our objective of determining the developmental dynamics of the *Dnmt3 *genes in postnatal oocytes summarized in Figure 6, we also wished to investigate whether the expression of these transcripts changed in response to the depletion of DNMT3L or DNMT1o. We chose to compare expression profiles after depletion of these two DNMTs in particular because the former provides a mouse model where maternal methylation imprints are affected [14], while the latter represents a model where oocyte methylation imprint patterns are unchanged [8]. Again, we used QRT-PCR to look at the expression of *Dnmt3a *and *Dnmt3b *in oocytes isolated from heterozygous and homozygous *Dnmt3L *females at 15 dpp (Figure 4a) and 25 dpp (see Additional file 3). Expression results were calibrated to the value obtained for the heterozygous oocytes and normalized to rabbit α-globin transcript levels. When compared to the heterozygous oocytes transcript levels, *Dnmt3a *and *Dnmt3b *relative expression was up-regulated in response to DNMT3L depletion, increasing roughly 2- and 3-fold respectively. The increase in *Dnmt3b *expression was highly significant (p < 0.001) while the increase in *Dnmt3a *expression only approached significance but was reproducible. We also analyzed *Dnmt3L *expression levels and found heterozygous 15 dpp oocytes to have 360 times the transcript levels as homozygous mutant 15 dpp oocytes (p < 0.01), confirming the purity of our samples (Figure 4b). Analysis of *Dnmt3a *and *Dnmt3b *expression changes as a result of DNMT3L depletion in 25 dpp GV oocytes also showed up-regulation of *Dnmt3a *and *Dnmt3b*, although not to the same degree as that seen at 15 dpp (see Additional file 3).

**Figure 5:**
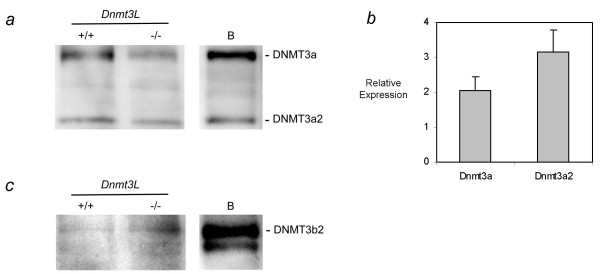
**Protein and transcript variant analysis of *Dnmt3a *and *Dnmt3b *in DNMT3L wild type and depleted 15 dpp oocytes**. Western Blot analysis of **a) **DNMT3A and **c) **DNMT3B was carried out on growing oocytes isolated from *Dnmt3L *wild-type and homozygous 15 dpp females (280 oocytes/lane). For comparison and isoform identification, the panels on the right labelled 'B' show blotting results for type B spermatogonia (for the loading control see Additional file 4). As suggested by the RNA expression analysis in (b), both DNMT3a and DNMT3a2 are present in growing oocytes and the ratio of these transcripts does not change significantly in *Dnmt3L *homozygous oocytes. DNMT3b2 is the more abundant isoform in wild-type *Dnmt3L *oocytes and appears to be upregulated in mutant oocytes as also suggested by the RNA expression data in Figure 4a. **b) **Relative expression of Dnmt3a and Dnmt3a2 was determined using transcript specific primers. Expression was normalized to 18S. As previously mentioned, both transcripts are expressed in growing oocytes.

These changes at the RNA expression level were also investigated by Western Blot analysis for DNMT3A and DNMT3B in 15 dpp wild-type and homozygous *Dnmt3L *oocytes (Figure 5a,c). This also allowed us to examine which isoforms of the *de novo *methyltransferases are present in growing oocytes. As shown in Figure 5a, both DNMT3a isoforms, DNMT3A and DNMT3A2, were detected in 15 dpp growing oocytes. QRT-PCR analysis also showed the transcript variants of *Dnmt3a *to be of similar abundance in growing oocytes (Figure 5b). Although we saw an increase in *Dnmt3a *expression in DNMT3L depleted oocytes, a comparable increase in the amount of either DNMT3a isoform in the absence of DNMT3L was not observed when compared to the loading control (see Additional file 4). In contrast, the significant increase in expression found for *Dnmt3b *in *Dnmt3L *-/- oocytes was also seen at the protein level (Figure 5c). It appears that the major isoform of DNMT3b in growing oocytes is DNMT3b2, since the band migrates to the same height as the main isoform in type B spermatogonia [25].

**Figure 6:**
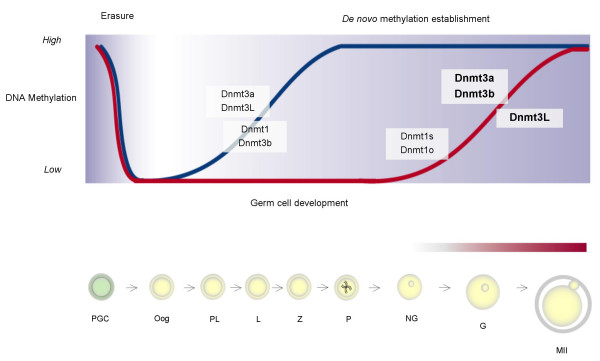
**Onset of DNA methyltransferase expression during mammalian germ cell development**. The results presented in this paper as well as observations made for the male germ line [25, 26] are depicted here in relationship to the timing of *de novo *methylation establishment on imprinted genes and repeats during germ cell development. The figure is adapted from [47]. The top panel illustrates the methylation dynamics of maternally and paternally methylated imprinted genes, depicted by the red and blue lines respectively. During gametogenesis the pattern of non-imprinted gene methylation closely resembles that of imprinted genes. The time at which DNA methyltransferases are expressed during germ cell development are indicated with the transcripts examined in this study highlighted in bold. Although expressed at similar stages of oogenesis, the *de novo *DNA methyltransferases, Dnmt3a and Dnmt3b, are grouped separately from Dnmt3L which does not have DNA methyltransferase activity. The onset of Dnmt1o expression has been previously shown in early growing oocytes [43]. Our findings show that the expression of *Dnmt3a*, *Dnmt3b *and *Dnmt3L *coincide with the establishment of DNA methylation on imprinted genes and repeat sequences in the female germ line. The progression of methylation imprint acquisition during oogenesis is illustrated in the bottom panel and depicted by the red shading above the female germ cells. PGC = primordial germ cell, Oog = oogonia, PL = preleptotene, L = leptotene, Z = zygotene, P = pachytene, NG = non-growing oocyte G = growing oocyte, MII = metaphase II oocyte.

We next looked at the changes in expression of *Dnmt3a*, *Dnmt3b*, and *Dnmt3L *in 15 dpp and 25 dpp oocytes isolated from *Dnmt1o *heterozygous and homozygous mice and found a similar pattern of transcriptional up-regulation seen in the case of DNMT3L depleted oocytes. Two independent experiments each performed in triplicate on two different pools of both heterozygous and homozygous oocytes showed that while Dnmt3L transcript levels were not affected, the *de novo *DNMTs, *Dnmt3a *and *Dnmt3b*, showed an increase in relative expression, with a roughly 1.5 and 2 fold increase respectively in 25 dpp homozygous oocytes (representative results from one experiment are depicted in Figure 4c). Dnmt3b was consistently up-regulated to a greater extent than Dnmt3a at all time points and as a result of either DNMT1o or DNMT3L depletion. At 15 dpp, no reproducible change in Dnmt3a, Dnmt3b or Dnmt3L expression levels were observed in DNMT1o null oocytes (see Additional file 5). We confirmed the purity of our germ cell preparations by determining Dnmt1o expression levels in both heterozygote and homozygote DNMT1o deficient oocytes and found expression in the heterozygote oocytes to be roughly 44 times that seen in homozygote oocytes (data not shown).

## Discussion & conclusion

The establishment and maintenance of DNA methylation patterns on imprinted genes and repeated sequences are essential for normal gametogenesis and embryogenesis in mammals. However, the developmental expression dynamics of the DNMT enzymes responsible for establishing and maintaining these DNA methylation patterns are not well understood, particularly in female germ cells. In the present study, we used QRT-PCR to determine the expression profiles of *Dnmt3a*, *Dnmt3b *and *Dnmt3L *in postnatal oocytes during the period when methylation patterns are being established. We also examined whether expression of these transcripts is related to oocyte diameter, investigated the transcript variants and protein isoforms of *Dnmt3a *and *Dnmt3b *found in growing oocytes, and determined the effect of depleting two DNMT enzymes, DNMT1o and DNMT3L, on the relative expression of *Dnmt3a*, *Dnmt3b *and *Dnmt3L*. In addition to exploring the expression dynamics of the *de novo *enzymes in *Dnmt3L *homozygous oocytes, we also investigated the methylation defects in these oocytes, with a particular interest in determining the effect of DNMT3L depletion on the methylation status of retrotransposons in female germ cells.

### Developmental profile of Dnmt3a, Dnmt3b, and Dnmt3L during postnatal oocyte growth and comparison to their expression during spermatogenesis

Several reports have provided some detail on the expression of *Dnmt3a*, *Dnmt3b *and *Dnmt3L *in mouse oocytes. The staining of adult ovaries with X-Gal to reveal the accumulation of a β-geo marker driven by the endogenous *Dnmt3L *promoter showed *Dnmt3L *to be accumulated in growing oocytes [14,15]. We have previously used semi-quantitative RT-PCR to determine the expression profile of these transcripts during postnatal female germ cell development and showed that they are expressed in growing oocytes [19]. QRT-PCR has been used to determine the expression patterns of these DNMTs in the ovary, and while *Dnmt3a *and *Dnmt3b *expression appeared to be stable across the time points examined, *Dnmt3L *expression clearly peaked post-natally [26]. Still, because of the large population of somatic cells in the ovary, a more thorough assessment of the expression of *Dnmt3a*, *Dnmt3b *and *Dnmt3L *in isolated female germ cells remained necessary to better understand the developmental profile of these DNMTs during oogenesis. Our results suggest that *Dnmt3a*, *Dnmt3b *and *Dnmt3L *have similar expression profiles, with their expression peaking during the crucial period in postnatal oocyte development when maternal methylation imprints are established [19]. Analysis of the differences between the relative expression of the DNMTs suggests that *Dnmt3L *and *Dnmt3a *are the most highly upregulated enzymes, consistent with their essential role and cooperation in establishing maternal methylation imprints [14,15,20,27]. The increased sensitivity offered by using QRT-PCR has allowed us to detect Dnmt3a, Dnmt3b and Dnmt3L transcripts in MII oocytes which we did not observe in our preliminary analysis [19]. This raises the possibility that these enzymes are stored as maternal transcripts and may play a role in *de novo *and/or maintenance methylation during preimplantation development.

In addition to studies focusing on female germ cells, the expression of these enzymes in mice has also been investigated during murine spermatogenesis. Depletion of DNMT3a and DNMT3L in male germ cells leads to impaired spermatogenesis [14-16,20]. Expression of *Dnmt3L *appears to be confined to a brief perinatal period during which it is highly expressed in non-dividing spermatogonial stem cell precursors [13]. The testis-wide and germ cell specific expression of the DNMT3 transcripts has been examined using a QRT-PCR approach [25,26]. In the developing testis, *Dnmt3a *and *Dnmt3L *have similar expression profiles with expression peaking pre-natally at 15.5 dpc, while *Dnmt3b *expression peaks post-natally at 6 dpp [26].

Comparing the developmental expression profile of *Dnmt3a*, *Dnmt3b *and *Dnmt3L *in oocytes and the expression patterns previously described in the male germ line, sex-specific differences in the expression of this family of enzymes are apparent (Figure 6). The perinatal expression of both *Dnmt3a *and *Dnmt3L *in male germ cells fits well with the timing of methylation pattern establishment during spermatogenesis. Both paternally methylated imprinted genes and repeat sequences begin to acquire methylation pre-natally in prospermatogonia and DNA methylation patterns continue to be established and maintained peri-natally [18,28-30]. In contrast, *Dnmt3a*, *Dnmt3b *and *Dnmt3L *are expressed in postnatal growing oocytes during the period when maternally methylated imprinted genes and repeat elements acquire methylation [19,23,24,30,31].

### Increased Dnmt3a, Dnmt3b, Dnmt3L and Dnmt1o expression correlated with an increase in oocyte diameter

We have previously shown that methylation acquisition within the DMR of at least one imprinted gene is related to oocyte diameter [19]. Hiura et al. (2006) [24] and Bao et al. (2000) [23] also found that a certain threshold in oocyte diameter is necessary for epigenetic modifications to have been thoroughly established in oocytes. Preliminary semi-quantitative RT-PCR analysis suggested to us that the expression of *Dnmt3L *may be influenced by oocyte diameter [19]. Here, using a QRT-PCR approach we confirmed *Dnmt3L *expression to be correlated to oocyte diameter and also found *Dnmt3a*, *Dnmt3b *as well as *Dnmt1o *expression to significantly increase with increasing oocyte diameter. Postnatal oogenesis is a period during which the oocyte grows greatly in volume and accumulates a variety of factors necessary for meiotic maturation and early preimplantation development [32]. The increase in transcription that accompanies this growth period also leads to elevated expression levels of *Dnmt3a*, *Dnmt3b*, *Dnmt3L *and *Dnmt1o *in fully grown oocytes.

### Aberrant methylation of Snrpn, Peg3, and Igf2r but normal methylation of IAP and LINE-1 retrotransposons in growing Dnmt3L-/- oocytes

It has been previously reported that depletion of DNMT3L in the oocyte results in impaired establishment of DNA methylation patterns on imprinted genes inheriting a maternal methylation mark [14,15]. Although abnormal methylation of the *Snrpn *and *Peg1 *DMRs was described in mature MII oocytes [14], it remained unknown if different DMRs showed the same methylation defect and if this defect occurred early or late during female germ cell development. Here, we analyzed the methylation profile of *Snrpn*, *Igf2r*, and *Peg3 *in DNMT3L deficient mid-growing oocytes. We have previously observed methylation levels on these 3 genes at 15 dpp to be between 60 and 80% [19]. In keeping with the previously reported *Snrpn *and *Peg1 *results [14], we found *Snrpn*, *Peg3 *and *Igf2r *to be unmethylated in *Dnmt3L*-/- 15 dpp oocytes, suggesting that DNMT3L is involved in the earliest stages of maternal methylation imprint acquisition.

In addition to analyzing imprinted gene methylation, we also examined the methylation status of the IAP 5' LTR and the LINE-1 5' UTR in both growing and mature oocyte samples and found these retrotransposable elements to be normally methylated when compared to prior analysis of these same regions in oocytes [19,33]. While the methylation status of retrotransposons in *Dnmt3L *homozygous oocytes was unknown, previous experiments suggested these sequences to be unaffected in post-implantation *Dnmt3L*+/- embryos derived from the fertilization of DNMT3L deficient oocytes [14]. Detailed studies have clearly shown that DNMT3L is required for establishing methylation on LTR and non-LTR retrotransposable elements in the male germ line and that this role for DNMT3L is important for ensuring normal meiosis [13,16]. In contrast, our analysis indicates that DNMT3L is not required for the establishment of methylation patterns on IAPs or LINE-1s in oocytes. In contrast to sperm, IAP and LINE-1 elements are not fully methylated in mature wild-type oocytes. This may be additional evidence of some different requirement of the two parental germ lines on methylation-dependent retrotransposon silencing. Together, these observations suggest germ line specific functions for DNMT3L and demonstrate that its involvement in retrotransposon silencing is restricted to male germ cells. Analysis of IAP LTR methylation in DNMT3a depleted oocytes showed these repeats to be hypomethylated [20]. It remains possible that other factors together with DNMT3a are involved in retrotransposable elements methylation during oocyte development. Interestingly, a recent report suggests the involvement of LSH, a member of the SNF2-helicase family of chromatin remodellers, in retrotransposon silencing in female germ cells [34].

### Up-regulation of the de novo DNMTs in DNMT1o and DNMT3L depleted oocytes

In contrast to the *Dnmt3L *knockout, where genomic imprints are not established [14], depletion of DNMT1o does not affect the acquisition of genomic imprints [8]. To determine whether *Dnmt3a*, *Dnmt3b *and *Dnmt3L *are coordinately regulated or if they can compensate for each other, the expression profiles of these enzymes were compared between heterozygous and homozygous oocytes for both models. QRT-PCR results indicated that in response to DNMT1o depletion, the expression of the *de novo *DNMTs, *Dnmt3a *and *Dnmt3b*, was up-regulated in 25 dpp GV oocytes, while *Dnmt3L*, which lacks DNA methyltransferase activity, showed no significant change. *Dnmt3a *and *Dnmt3b *were also up-regulated in the 15 dpp *Dnmt3L*-/- oocytes analyzed. While the increase in *Dnmt3a *expression was modest, *Dnmt3b *expression was significantly upregulated in both DNMT1o and DNMT3L depleted oocytes with a parallel increase in protein expression observed in DNMT3L depleted 15 dpp oocytes. Similarly, Gius et al. (2004) [35] also observed a three-fold increase in Dnmt3b transcripts in a *Dnmt1 *knockout cell line. These findings suggest that in the absence of one DNMT, a compensatory feedback mechanism may exist which leads to the up-regulation of *Dnmt3b *in an attempt to rescue the defect in *de novo *and/or maintenance methylation.

It is possible that while such an upregulation may play a role in maintaining DNA methylation on retrotransposons and other repeat sequences in the genome of *Dnmt3L*-/- mice, it is unable to maintain methylation on imprinted sequences due to the absence of DNMT3L. The activation of retrotransposons in the male germ line has been shown to have deleterious consequences on meiosis [13]. IAP transposons also appear to be resistant to active demethylation 'reprogramming' in primordial germ cells and zygotes [36]. In this report the authors speculate that maintaining these elements methylated may prevent their transposition and consequently avert the introduction of deleterious mutations in the genomes of gametes and preimplantation embryos. Therefore, the upregulation of DNMTs may be an additional mechanism which has evolved to ensure the silencing of these elements in the female germline. However, upregulation of a DNMT not normally expressed at high levels at a certain stage of oocyte development could potentially also have negative repercussions and contribute to aberrant methylation patterns in *Dnmt *knock-out oocytes.

Recent analysis of stochastic imprinting events in the progeny of *Dnmt3L*-/- females showed that sporadically, in some embryos derived from DNMT3L null oocytes, maternal DMRs were methylated and imprinted expression was normal [37]. The authors suggest that in the absence of DNMT3L, the other DNMTs or additional factors may act to rescue imprint establishment in the germline [37]. It is possible that the up-regulation of *Dnmt3b *that we observed in *Dnmt3L*-/- growing oocytes may contribute to this rescue. Interestingly, for *Igf2r*, one of the sequenced molecules was methylated at 6 of the 7 CpG sites analyzed (Figure 3a). This observation may be reflective of the stochastic imprinting event also observed by Arnaud et al. and may be a result of the rare instance where the upregulation of the other DNMTs was able to rescue the methylation imprint. The overall failure of the DMRs to be methylated during oogenesis in *Dnmt3L*-/- oocytes which are 'over-expressing' *Dnmt3b *may be due to the inefficient targeting of the enzymes in the absence of DNMT3L. Previous data have shown maternally imprinted genes in *Dnmt3L *-/- mature MII oocytes to be unmethylated [14,15]. Therefore an alternative possibility to reconcile the results presented here with those described by Arnaud et al. is that in the absence of DNMT3L the DMRs acquire methylation during preimplantation development perhaps as a result of increased expression of *Dnmt3a *and *Dnmt3b *which are carried over as maternal transcripts.

Prior studies have pointed to the interaction and interdependence of DNMT enzymes. *In vitro *studies have suggested that DNMT3L can stimulate *de novo *methylation catalyzed by DNMT3a at several ICEs [27]. Physical interactions between DNMT3L and the C-terminal domain of both DNMT3a and DNMT3b, as well as interactions between DNMT1 and DNMT3a or DNMT3b have been described [38-40]. Together with these studies, the QRT-PCR results described here suggest that *Dnmt3a*, *Dnmt3b *and *Dnmt3L *are developmentally and may be coordinately regulated to ensure the proper methylation of both imprinted and non-imprinted sequences in the female germ line.

## Methods

### Oocyte collections and mice

MII oocytes as well as growing oocytes at 5, 10, 15, 25 dpp were isolated as previously described [19,41]. Briefly, oocytes at 5, 10, 15, and 25 dpp were isolated from dissociated ovaries obtained from CD-1 mice (Charles River Canada, St. Constant, QC). The dissection of 5, 10, and 15 dpp ovaries was carried out in PBS, pH 7.2. After transfer to conical tubes containing 2 ml of 3 mg/ml polyvinylpyrrolidone (Sigma) prepared in PBS, 2 mg/ml collagenase (Sigma), 0.025% trypsin (Gibco BRL) and 0.02 mg/ml DNase (Sigma), the contents were shaken in a 37°C incubator at 250 rpm for 3 to 10 minutes. The dissociated ovary mixture was then diluted by half with Hepes-buffered MEM, pH 7.2 (Gibco BRL), modified as described [42]. A micropipette was used to draw the media solution in and out to allow further dissociation of any oocyte-cumulus cell complexes. Twenty-five dpp GV stage oocytes were isolated by puncturing ovarian follicles and for oocytes at this time point 50 μg/ml dibutyryl cyclic AMP was added to the MEM-H in order to prevent GVBD. MII oocytes were collected from 7–8 week old CD-1 females superovulated by injection of 7.5 IU of pregnant mares' serum gonadotropin (Sigma). This first injection was followed 44–48 hours later by 5 IU of human chorionic gonadotropin, (Sigma). MII oocytes were recovered 20 hours post-hCG from the oviducts and 1 mg/ml hyaluronidase was used to disperse cumulus cells (Sigma).

All oocytes were collected using a mouth-controlled drawn-out glass pipette and somatic cells were removed by transferring all oocytes through three dishes of media. Only cumulus-free and non-fragmented oocytes were chosen for experiments. Oocytes were carefully collected for all samples so that the proportion of 20 to 50 μm and 60 to 80 μm oocytes were similar across replicates and also reflective of the proportion of oocytes found in the ovaries at respective stages. To verify size each oocyte was measured using an eyepiece micrometer. At 5 dpp oocytes measured 10–20 μm in diameter and were obtained from non-growing primordial follicles; 10 dpp oocytes were collected from a pool of early-growing follicles ranging greatly from 20 to 80 μm in diameter with roughly half measuring under 50 μm; while 95% of oocytes collected at 15 dpp measured 60 to 80 μm in diameter. GV stage oocytes which are fully grown (80 μm) were collected at 25 dpp and superovulated 8 week old females were used for the collection of ovulated MII oocytes.

Because of previously described differences in the transcript levels in oocytes of different diameters, particular attention was taken in collecting *Dnmt1o*- and *Dnmt3L*- wild-type, heterozygous and homozygous oocytes to ensure that equivalent pools of oocytes were collected for the different genotypes. After washing, oocytes were measured a second time to ensure that any observed expression differences were reflective of the genotype of the oocytes and not differences in their diameters. All experiments were conducted in compliance with Canadian Council for Animal Care guidelines.

Before storage at -80°C, 10 μl of Trizol (Invitrogen) was added to oocyte samples intended for RNA extraction to prevent RNA degradation. *Dnmt1o *and *Dnmt3L *+/- and -/- oocytes were collected using the same techniques outlined above from mice that have been previously described [8,14].

### Real-time RT-PCR

Oocyte total RNA was extracted using Trizol reagent according to the manufacturer's instructions and supplemented with 10 μg of mussel glycogen (Boehringer Ingelheim) as previously described [19,43]. To serve as an internal control for RNA extraction and amplification efficiency, 0.125 pg of rabbit α-globin mRNA (Sigma) was added per oocyte for each sample prior to RNA extraction [21]. Oocyte RNA pellets were dissolved in RNase-free water to give a final concentration of 10 oocytes per μl.

Total RNA was extracted from two independent collections of pooled oocytes for each QRT-PCR experiment performed. QRT-PCR was performed as previously described [26]. Briefly, experiments were carried out using the Quantitect SYBR Green RT-PCR kit (Qiagen) and performed on the Mx4000 QPCR system (Stratagene) using the standard curve method [44]. The primers used to amplify *Dnmt1o*, *Dnmt3a*, *Dnmt3b*, *Dnmt3L*, *18S *and rabbit α-*globin *have been previously described [19,26,43]; the primers used to assess expression of *Dnmt3a *and *Dnmt3b *were designed to estimate the overall expression of these genes. Expression of *Dnmt3a *was further explored using transcript variant-specific primers designed to determine the relative expression levels of Dnmt3a and Dnmt3a2, the two major transcripts of this gene. Primer sequences and amplification conditions are described in La Salle and Trasler, 2006 [25]. The primers used to determine the expression of the *Dnmt3a *transcript variants by QRT-PCR analyzed the expression of each individual variant: the sense primer was unique to each transcript while the reverse primer was common to both; the expression of each variant was assessed individually, not in the same reaction tube, to give an expression profile for each variant (and not a profile for Dnmt3a and a combined profile for Dnmt3a/Dnmt3a2).

QRT-PCR was carried out using 5 to 20 oocytes per sample depending on the *Dnmt *gene being tested and performed in triplicate for each of the 2 independent collections of oocytes. For the developmental studies, standard curves for each experiment were established using single-use aliquots of 6 dpp testes total RNA supplemented with rabbit α-globin, while standard curves for the studies involving the *Dnmt1o *and *Dnmt3L *+/- and -/- oocytes were established using single-use aliquots of 20 dpp ovary RNA also supplemented with rabbit α-globin. Data were analyzed by normalizing the RNA quantity determined for the test enzyme to the rabbit α-globin content of the same sample and plotted by calibrating to the lowest-expressing sample. Rabbit α-globin Ct values were consistent across samples and replicates suggesting that our mRNA recovery rate and cDNA synthesis efficiency was uniform across experiments. Single product amplification was verified by electrophoresing QRT-PCR products through 2% agarose gels. Results are presented as mean ± SD.

### DNA isolation and methylation analysis

DNA was isolated from roughly 500 *Dnmt3L*-/- oocytes at 15 dpp and bisulfite treatment was carried out as previously described [8,45,46]. Nested PCR amplification for *Snrpn*, *Igf2r*, *Peg3*, *H19*, and non-nested PCR amplification for IAPs were carried out also with bisulfite specific primers and conditions that have been previously described [8,45]. An ABI 310 sequencer was used to sequence clones containing the appropriate inserts and only sequences that showed a bisulfite conversion efficiency of >95% were used for analysis. Sequence differences between clones with similar CpG methylation profiles were verified to ensure unique clones were represented. We examined a total of 16 CpG sites in a 419 bp fragment of *Snrpn *(2151–2570 bp, **AF081460**), 7 CpG sites in a 205 bp fragment of *Igf2r *(796–1001 bp, **L06446**), 18 CpG sites in a 286 bp fragment of *Peg3 *(2770–3056 bp, **AF105262**), 16 CpG sites in a 422 bp fragment of *H19 *(1304–1726 bp, **U19619**) and 9 CpG sites in a 212 bp fragment of IAP (100–312 bp, **M17551**). Methylation analysis of *H19 *served as an internal control for somatic cell contamination as this imprinted gene acquires methylation during spermatogenesis and should be unmethylated in oocyte samples.

For IAP and LINE-1 methylation analysis, approximately 4 ng DNA, corresponding to approximately 700 oocytes, was digested with methylation sensitive restriction enzyme HpaII and methylation insensitive isoschizomer MspI before DNA blotting with IAP long terminal repeat (LTR) or LINE-1 5' UTR probes as previously described [13]. The experiment was done in duplicate with different oocyte samples and was reproducible.

### Protein extraction and immunoblotting

*Dnmt3L*-wild type and -homozygous oocytes collected at 15 dpp were boiled in reducing sample buffer for 5 minutes and electrophoresed on an 8% SDS-polyacrylamide gel. 280 oocytes were loaded per lane. A type B spermatogonia protein lysate prepared as previously described was also electrophoresed in parallel to serve as a positive control in the detection of DNMT3a and DNMT3b isoforms [25]. Proteins were transferred to a Hybond ECL nitrocellulose membrane (Amersham, Montreal, QC, Canada) that was blocked in 5% non-fat dried milk. The membrane was incubated with the following primary antibody diluted in blocking buffer: clone 64B1446, a monoclonal antibody (mAb) raised against recombinant mouse DNMT3a (1:400; Imgenex, San Diego, CA). The membrane was washed according to the manufacturer's instruction (Amersham) and incubated with a horseradish peroxidase (HRP)-conjugated goat anti-mouse IgG antibody (1:1000; Vector Laboratories). After exposure to ECL Plus Western Blotting detection solution (Amersham), chemiluminescence was revealed on Hyperfilm ECL film (Amersham). The membrane was then stripped and reprobed with the following primary antibody diluted in blocking buffer: clone 52A1018, a mAb raised against recombinant mouse DNMT3b (1:400; Imgenex). India ink staining of the membrane was used to confirm loading (see Additional file 4).

### Statistical analysis

SPSS software was used for statistical analysis of the QRT-PCR data obtained where the number of replicates allowed for valid interpretation. Prior to performing statistical tests, the data was analyzed to determine whether it conformed to a normal distribution. Comparisons between timepoints or samples were performed using a univariate analysis of variance. For multiple comparisons, Bonferroni or Games-Howell post hoc tests were used. A p-value of < 0.05 was taken to be significant.

## Abbreviations

DNMTs DNA methyltransferases

DMRs Differentially methylated regions

ICEs Imprinting control elements

QRT-PCR Real-time RT-PCR

MII Metaphase II

## Authors' contributions

DL carried out the oocyte isolations, QRT-PCRs, and bisulfite sequencing experiments unless otherwise indicated. SL was responsible for the *Dnmt3L *mice husbandry and genotyping, designed the QRT-PCR primers, optimized QRT-PCR cycling conditions, and performed the transcript-specific QRT-PCR and Western Blot analyses. DB performed the IAP Southerns. JM was responsible for the *Dnmt1o *mice husbandry and genotyping. THB participated in the study design. DL, SL and JMT were responsible for the study design and manuscript drafting. DB, THB and JM revised the manuscript and all authors read and approved the final manuscript.

## Supplementary Material

Additional file 1 **Developmental expression profile of 18S during postnatal oogenesis.** QRT-PCR was used to determine the expression profile of 18S in postnatal oocytes.  Relative expression values obtained were normalized to the level of rabbit α-globin expression for each sample.  18S expression significantly increased between 5 and 10 dpp (p<0.01) and again between 10 and 15 dpp (p<0.05) making it an unsuitable normalizer for experiments looking at the expression level of mRNA transcripts during postnatal oocyte development.  Results are presented as mean ± SD. Click here for file

Additional file 2 **Developmental expression profiles of *Dnmt3a*, *Dnmt3b*, and *Dnmt3L* during postnatal oogenesis â€“ second replicate.** QRT-PCR was used to determine the expression profile of **a)*** Dnmt3a *(light grey bars) **b)*** Dnmt3b* (cross hatch bars) and **c) ***Dnmt3L* (dark grey bars) in postnatal oocytes.  Relative expression values obtained were normalized to the level of rabbit α-globin expression for each sample and were calibrated to the MII oocyte expression value.  Results are presented as mean ± SD. For *Dnmt3a*, a significant increase in expression was observed between 5 and 10 dpp (p<0.05); while in the case of both *Dnmt3b* and *Dnmt3L*, the increase between 10 and 15 dpp was found to be significant (p<0.05). Click here for file

Additional file 3 ***Dnmt3a* and *Dnmt3b* expression in DNMT3L deficient 25 dpp oocytes.** QRT-PCR was used to analyze the expression of the *de novo* DNMT enzymes in *Dnmt3L* heterozygous (dark grey bars) and homozygous (cross hatch bars) GV stage oocytes at 25 dpp.  Dnmt3a and Dnmt3b transcripts are not significantly up-regulated in DNMT3L depleted oocytes at this time point. Samples were analyzed in triplicate and relative expression values obtained were normalized to the level of rabbit a-globin expression for each sample and were calibrated to the expression in heterozygous oocytes for *Dnmt3a* and *Dnmt3b*.  Results for one experiment are presented as mean ± SD. Click here for file

Additional file 4 **Loading control for DNMT3A and DNMT3B Westerns.** India ink staining of the membrane after probing and transfer was used to confirm equal loading for the samples shown in Figure 5.  Western Blot analysis of DNMT3A and DNMT3B was carried out on growing oocytes isolated from *Dnmt3L* wild-type and homozygous 15 dpp females (280 oocytes/lane).  The panel on the right labelled â€˜Bâ€™ shows blotting results for type B spermatogonia Click here for file

Additional file 5 ***Dnmt3a*, *Dnmt3b* and *Dnmt3L* expression in DNMT1o deficient 15 dpp oocytes.** QRT-PCR was used to analyze the expression of the DNMT enzymes in *Dnmt1o* heterozygous (dark grey bars) and homozygous (cross hatch bars) 15 dpp growing oocytes.  The relative expression of *Dnmt3a*, *Dnmt3b* and *Dnmt3L* was not significantly changed in DNMT1o depleted oocytes at this time point. Samples were analyzed in triplicate and relative expression values obtained were normalized to the level of rabbit a-globin expression for each sample and were calibrated to the expression in homozygous oocytes for *Dnmt3a*, *Dnmt3b* and *Dnmt3L*.  Results for one experiment are presented as mean ± SD. Click here for file
