# Supplementary figures and images for: Coordinate regulation of DNA methyltransferase expression during oogenesis
Source: BMC Dev Biol. 2007 Apr 19;7:36. doi: 10.1186/1471-213X-7-36 (PMC1878483; doi:10.1186/1471-213X-7-36)

## Slide 1
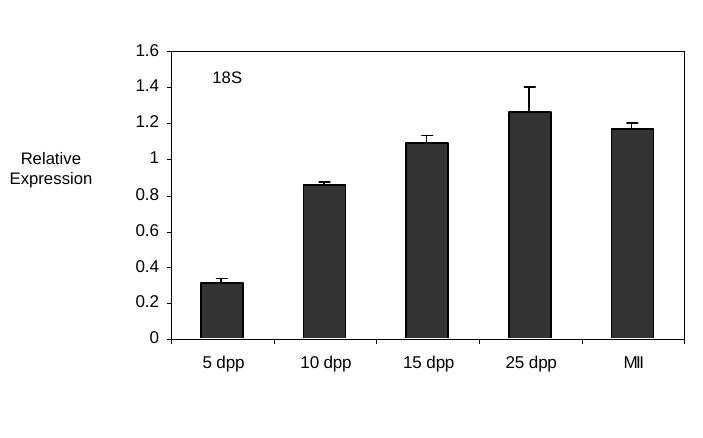

18S
Relative Expression

Supplement: Additional file 1 — Developmental expression profile of 18S during postnatal oogenesis. QRT-PCR was used to determine the expression profile of 18S in postnatal oocytes. Relative expression values obtained were normalized to the level of rabbit α-globin expression for each sample. 18S expression significantly increased between 5 and 10 dpp (p<0.01) and again between 10 and 15 dpp (p<0.05) making it an unsuitable normalizer for experiments looking at the expression level of mRNA transcripts during postnatal oocyte development. Results are presented as mean ± SD. [file 1471-213X-7-36-S1.ppt]

## Slide 1
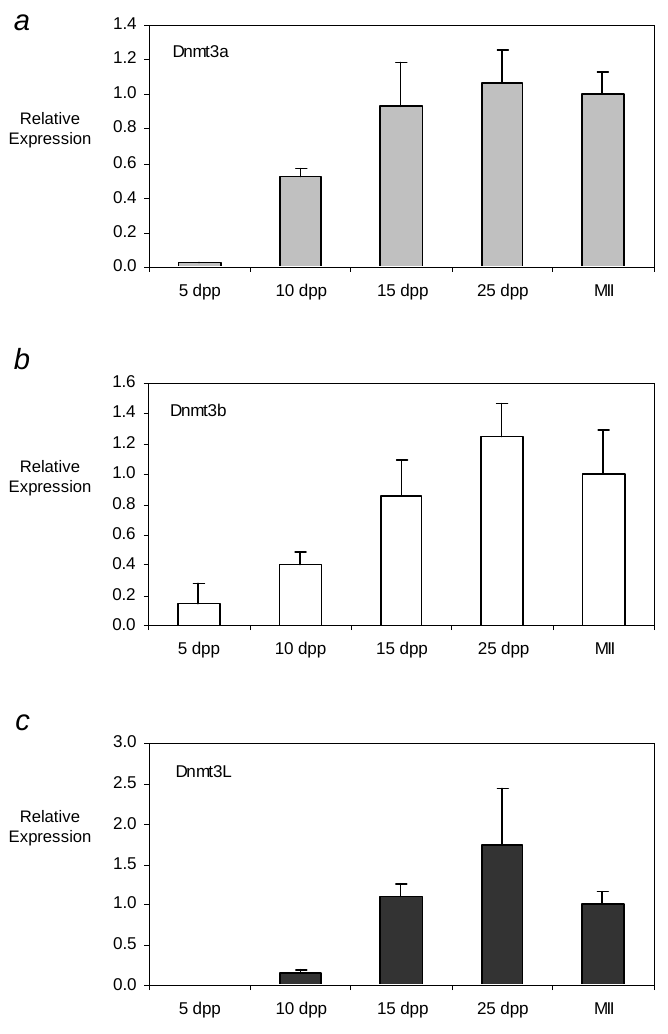

a
Relative Expression
b
Relative Expression
c
Relative Expression

Supplement: Additional file 2 — Developmental expression profiles of Dnmt3a, Dnmt3b, and Dnmt3L during postnatal oogenesis â€“ second replicate. QRT-PCR was used to determine the expression profile of a) Dnmt3a (light grey bars) b) Dnmt3b (cross hatch bars) and c) Dnmt3L (dark grey bars) in postnatal oocytes. Relative expression values obtained were normalized to the level of rabbit α-globin expression for each sample and were calibrated to the MII oocyte expression value. Results are presented as mean ± SD. For Dnmt3a, a significant increase in expression was observed between 5 and 10 dpp (p<0.05); while in the case of both Dnmt3b and Dnmt3L, the increase between 10 and 15 dpp was found to be significant (p<0.05). [file 1471-213X-7-36-S2.ppt]

## Slide 1
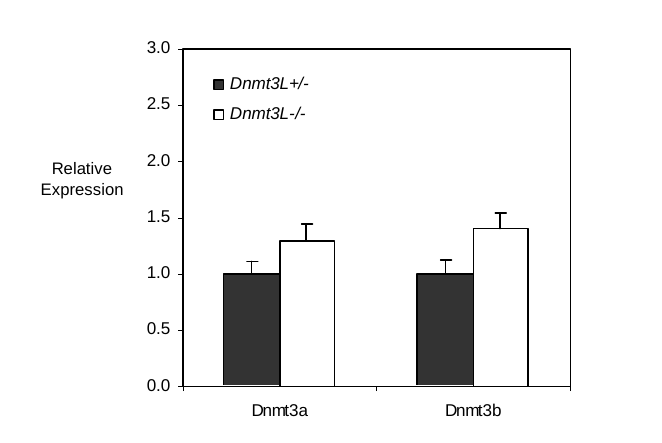

Relative Expression

Supplement: Additional file 3 — Dnmt3a and Dnmt3b expression in DNMT3L deficient 25 dpp oocytes. QRT-PCR was used to analyze the expression of the de novo DNMT enzymes in Dnmt3L heterozygous (dark grey bars) and homozygous (cross hatch bars) GV stage oocytes at 25 dpp. Dnmt3a and Dnmt3b transcripts are not significantly up-regulated in DNMT3L depleted oocytes at this time point. Samples were analyzed in triplicate and relative expression values obtained were normalized to the level of rabbit a-globin expression for each sample and were calibrated to the expression in heterozygous oocytes for Dnmt3a and Dnmt3b. Results for one experiment are presented as mean ± SD. [file 1471-213X-7-36-S3.ppt]

## Slide 1
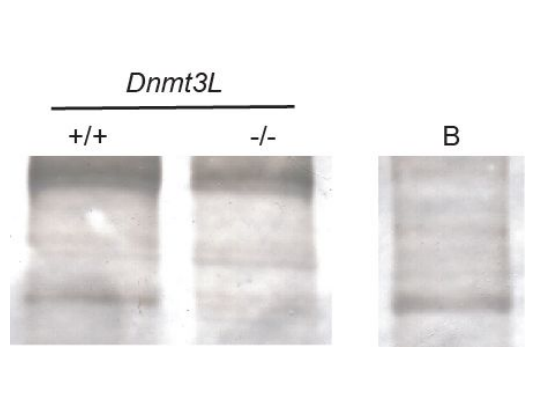

Supplement: Additional file 4 — Loading control for DNMT3A and DNMT3B Westerns. India ink staining of the membrane after probing and transfer was used to confirm equal loading for the samples shown in Figure 5. Western Blot analysis of DNMT3A and DNMT3B was carried out on growing oocytes isolated from Dnmt3L wild-type and homozygous 15 dpp females (280 oocytes/lane). The panel on the right labelled â€˜Bâ€™ shows blotting results for type B spermatogonia [file 1471-213X-7-36-S4.ppt]

## Slide 1
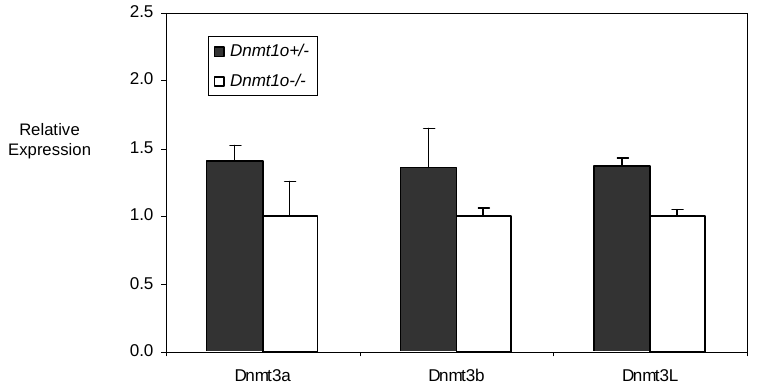

Relative Expression

Supplement: Additional file 5 — Dnmt3a, Dnmt3b and Dnmt3L expression in DNMT1o deficient 15 dpp oocytes. QRT-PCR was used to analyze the expression of the DNMT enzymes in Dnmt1o heterozygous (dark grey bars) and homozygous (cross hatch bars) 15 dpp growing oocytes. The relative expression of Dnmt3a, Dnmt3b and Dnmt3L was not significantly changed in DNMT1o depleted oocytes at this time point. Samples were analyzed in triplicate and relative expression values obtained were normalized to the level of rabbit a-globin expression for each sample and were calibrated to the expression in homozygous oocytes for Dnmt3a, Dnmt3b and Dnmt3L. Results for one experiment are presented as mean ± SD. [file 1471-213X-7-36-S5.ppt]
